# Whole genome resequencing and custom genotyping unveil clonal lineages in ‘Malbec’ grapevines (*Vitis vinifera* L.)

**DOI:** 10.1038/s41598-021-87445-y

**Published:** 2021-04-08

**Authors:** Luciano Calderón, Nuria Mauri, Claudio Muñoz, Pablo Carbonell-Bejerano, Laura Bree, Daniel Bergamin, Cristobal Sola, Sebastian Gomez-Talquenca, Carolina Royo, Javier Ibáñez, José Miguel Martínez-Zapater, Diego Lijavetzky

**Affiliations:** 1 Instituto de Biología Agrícola de Mendoza (IBAM, CONICET-UNCuyo), Almirante Brown 500, M5528AHB. Chacras de Coria, Mendoza, Argentina; 2 Instituto de Ciencias de la Vid y del Vino (CSIC, UR, Gobierno de La Rioja), Finca La Grajera Autovía del Camino de Santiago LO-20 Salida 13, 26007 Logroño, Spain; 3 Facultad de Ciencias Agrarias, Universidad Nacional de Cuyo. Almirante, Brown 500, M5528AHB. Chacras de Coria, Mendoza, Argentina; 4 Max Planck Institute for Developmental Biology, Max-Planck-Ring 9, 72076 Tübingen, Germany; 5 Vivero Mercier Argentina, Ruta 40 Km 3273, M5509, Perdriel, Mendoza, Argentina; 6 Plant Virology Laboratory, EEA Mendoza INTA, San Martin 3853, 5507, Luján de Cuyo, Mendoza, Argentina

**Keywords:** Agricultural genetics, Genetic markers, Genomics, Genotype, Plant genetics, Plant sciences, Plant breeding, Plant domestication

## Abstract

Grapevine cultivars are clonally propagated to preserve their varietal attributes. However, genetic variations accumulate due to the occurrence of somatic mutations. This process is anthropically influenced through plant transportation, clonal propagation and selection. Malbec is a cultivar that is well-appreciated for the elaboration of red wine. It originated in Southwestern France and was introduced in Argentina during the 1850s. In order to study the clonal genetic diversity of Malbec grapevines, we generated whole-genome resequencing data for four accessions with different clonal propagation records. A stringent variant calling procedure was established to identify reliable polymorphisms among the analyzed accessions. The latter procedure retrieved 941 single nucleotide variants (SNVs). A reduced set of the detected SNVs was corroborated through Sanger sequencing, and employed to custom-design a genotyping experiment. We successfully genotyped 214 Malbec accessions using 41 SNVs, and identified 14 genotypes that clustered in two genetically divergent clonal lineages. These lineages were associated with the time span of clonal propagation of the analyzed accessions in Argentina and Europe. Our results show the usefulness of this approach for the study of the scarce intra-cultivar genetic diversity in grapevines. We also provide evidence on how human actions might have driven the accumulation of different somatic mutations, ultimately shaping the Malbec genetic diversity pattern.

## Introduction

Clonal propagation is a common practice in perennial crops. In this kind of growing systems, the expected degree of genetic variability is low among clones within a given cultivar. However, intrinsic genetic events such as somatic mutations still occur and accumulate over time^1^. Grapevine (*Vitis vinifera* L.) cultivars are perennial crops, displaying highly heterozygous genotypes originated from sexual crossing and clonally propagated to preserve their productive traits^2^. Grapevine is among the top five fruit crops in terms of tons produced worldwide^3^ and it has a rather relatively small genome size (~ 480 Mb)^4^. These features make this species an attractive model for studying the impact of somatic mutations on the genetic diversity of clonal crops^5–7^. In this regard, there are many well-documented cases of somatic mutations affecting traits that are interesting from a productive standpoint, mainly related to berry color^8–10^, berry aroma^11^, cluster shape^12,13^ and reproductive development^14,15^. However, somatic mutations do not always have qualitative consequences, and quantitative effects have also been reported among clones^16,17^, even with the mutation identified at the nucleotide resolution level^18^. Nonetheless, somatic mutations might not have known phenotypic consequences; with these so-called ‘silent’ mutations still constituting a valuable source of genetic diversity^5,6^. Silent mutations are also relevant and can be used in marker-assisted selection programs^19,20^, or as genetic markers to study anthropically influenced processes shaping cultivars’ current genetic diversity patterns^1,21,22^.

Genetic variation in grapevines is notorious if different cultivars are compared^23,24^. However, studying the genetic diversity at the intra-cultivar level is more challenging. This difficulty is due to the low extant variability and because traditional markers, like SSRs and SNPs selected from inter-cultivar polymorphisms, have shown low efficiency in such approaches^25–28^. Recently, the increased accessibility to genome-wide scale sequencing technologies, has made possible to address the study of clonal genetic diversity more accurately^5,6,29,30^.

Herein we focused on Malbec cultivar, which prime name is Cot^31^. This cultivar has for long been well-appreciated for the elaboration of high-quality red wines^32^. According to the genetic evidence^33^ and historical records^32,34^, Malbec was originated from the outcross of Prunelard and Magdeleine Noir des Charentes cultivars in Southwestern France (Cahors region). Malbec was then introduced in Argentina (Mendoza province) during the 1850s^32,34^. In fact, this South American region has produced the largest amounts of Malbec wine for the past two decades^35^. Malbec presents a remarkable clonal phenotypic diversity^16,36^ and a great adaptation capacity, being successfully introduced under a wide range of agroecological conditions across Argentina^37^. However, little is known about Malbec clonal genetic diversity. Tracing back the clonal propagation history of this cultivar, human-driven processes that could have shaped the current pattern of genetic diversity can be detected. In first place, the single seedling that was selected to be cultivated after the above-mentioned outcrossing. In second place, the “bottleneck effect” suffered when Malbec grapevines were initially introduced from France to Argentina. Finally, the accumulation of different somatic mutations on both sides of the Atlantic Ocean, as consequence of clonal propagation under different environmental pressures and selection criteria.

In this work we surveyed Malbec intra-cultivar genetic diversity, with emphasis on how anthropic actions might have driven the accumulation of different somatic mutations, propelling the origin of clonal lineages. We implemented a whole-genome resequencing (WGR) approach to detect single-nucleotide variants (SNVs). We focused specifically on SNVs, because we aimed at designing a high throughput genotyping experiment based on this type of genetic markers. After a stringent variant calling and validation process, a reduced set of SNVs was selected to custom-design a genotyping chip. The results obtained with this experiment provided information on Malbec clonal genetic diversity across an extensive sampling.

## Results

### The variant calling of four Malbec accessions provides insight on the intra-cultivar genetic diversity

We performed the WGR of four Malbec clonal accessions: MB53, MB59, C225 and C143, which differed from each other in their time span of clonal propagation in Argentina. In total, ~ 90 million paired-end reads per clone were produced, adding 45 Gb of genomic data (Supplementary Table S1). Filtered reads were aligned to the nearly homozygous *Vitis vinifera* L. reference genome PN40024^4^ (hereafter: PN40024), covering ~ 78% of its length, with a mean read depth of ~ 30× (Supplementary Table S1).

**Figure 1:**
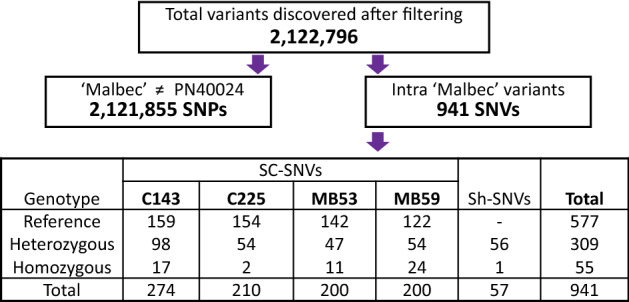
Total single nucleotide polymorphisms (SNPs) and single nucleotide variants (SNVs) identified in Malbec. SNPs were defined as variants that were common to the four clones, distinguishing Malbec from PN40024, whereas SNVs occurred differentially among the resequenced clones. SNVs were classified according to the clone for which they were identified and according to their genotype relative to PN40024. SC-SNVs are markers of variation in a single clone and Sh-SNVs are variants shared between two clones.

After variant calling and filtering, we detected a total of 2,122,796 variants (Fig. 1). More precisely, we found 2,121,855 single nucleotide polymorphisms (SNPs), defined as common variants to the four clones, differentiating Malbec from PN40024. We also identified 941 single nucleotide variants (SNVs), defined as variants distinguishing Malbec clones from each other. In average 98,501 SNPs and 47 SNVs per chromosome were observed (Supplementary Fig. S1). Most of the identified SNPs and SNVs affected intergenic regions, followed by variants affecting intronic and exonic regions (Supplementary Fig. S1 and Supplementary Table S2). We also observed that the number of transitions was higher than that of transversions; this was particularly noticeable for SNVs occurring in exonic regions, where transitions outnumbered transversions (Supplementary Table S2).

Out of 941 SNVs, 884 were single clone variants (hereafter: SC-SNVs); this is, each clone had a genotype that was different from the other three. On the other hand, 57 SNVs were shared (hereafter: Sh-SNVs); this is, two clones had the same genotype and different from the other two clones. Genotypes for SC-SNVs were classified in relation to the reference genome as heterozygous (one clone having a heterozygous alternative allele, while the other three remained homozygous as the reference, 253 SC-SNVs), reference (one clone showed the reference allele in homozygosis and the other three shared the same alternative heterozygous allele, 577 SC-SNVs), and homozygous (one clone with an homozygous alternative allele and the other three clones were either heterozygous alternative or homozygous reference, 54 SC-SNVs) (Fig. 1). Sh-SNVs genotypes were defined as either heterozygous or homozygous when two clones shared the same alternative allele in either of those states. Only one single Sh-SNV was homozygous, which was shared by C143 and C225, this position turned heterozygous for MB53 and MB59. The remaining Sh-SNVs were heterozygous and were distributed as follows: 17 were shared by MB53-MB59 and 17 by C143-C225, the other 22 Sh-SNVs were shared in different combinations: C143-MB53 (n = 3), C225-MB59 (n = 4), C225-MB53 (n = 6) and C143-MB59 (n = 9).

**Figure 2:**
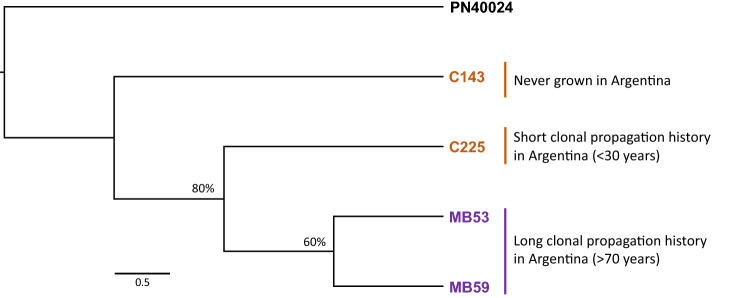
Phylogenetic relations among four resequenced Malbec accessions. Neighbor-joining tree based on *p*-distances and employing 941 SNVs. Percentages on the nodes represent bootstrap supports after 200 iterations, values > 50% are shown. The PN40024 genotype was used as outgroup.

We performed a phylogenetic analysis based on the 941 SNVs using PN40024 as an outgroup, and observed that the genetic relations among the four resequenced accessions were associated with their time span of clonal propagation in Argentina (Fig. 2). More precisely, C143, which has never been grown in Argentina, was the most genetically divergent clone, as compared to the other three. On the other hand, C225, which has a short history of clonal propagation in Argentina (< 30 years), differentiated (80% bootstrap support) from MB53 and MB59. Finally, MB53 and MB59, the two clones that have been propagated in Argentina for a longer period of time (> 70 years) also proved to be divergent (60% bootstrap support), but more closely related to each other than to the other two clones.

Out of the 941 described SNVs, 34 were chosen for validation by Sanger sequencing (Supplementary Table S3). This selection was based mainly on the capacity of SNVs to differentiate among the four resequenced accessions. We also considered the characteristics of the sequence surrounding the SNV, that could have affected the primers design and the genotyping experiment (see “Methods”). All the sequenced SNVs presented the expected allelic states for the corresponding clone, demonstrating the reliability of the bioinformatics procedures employed. Electropherogram alignments of four validated SC-SNVs are shown in Supplementary Fig. S2.

### The genotyping analysis suggests the existence of two Malbec clonal lineages

The genetic diversity was evaluated through a custom-designed genotyping chip consisting of 48 SNVs and built on the basis of the 34 validated SNVs. The chip included 42 SC-SNVs and 6 Sh-SNVs (Supplementary Table S4). The criteria for selecting the other 12 SNVs were the same as those previously mentioned for validation, this is further described in the “Methods”. Final analyses of genetic diversity were performed on 214 successfully genotyped Malbec accessions and based on 41 properly working SNVs (37 SC-SNVs and 4 Sh-SNVs). Seven out of the 48 starting SNVs and five out of the 219 starting samples were excluded due to technical problems related to missing data. Based on the resequenced clone for which each SNV was originally identified, the 37 properly working SC-SNVs were distributed as follows: nine for C143, seven for C225, 11 for MB53 and 10 for MB59; while the four Sh-SNVs corresponded to heterozygous variants shared by MB53 and MB59. Regarding the 41 SNVs variability, as expected for de novo mutations, most of them were transitions and only eight were transversions (transitions/transversions ratio = 4.1). Among the properly working genetic markers, 22 SNVs proved particularly informative across the studied clonal population, as they ranged between two and 164 samples that showed the alternative heterozygous allele (Supplementary Table S5). Only one of these markers (C225-snv4) showed the three possible genotypes, including the alternative allele in homozygosis. The remaining 19 SC-SNVs showed the alternative heterozygous allele only for one out of the four resequenced clones, which were analyzed with the genotyping chip as a proof of concept of its precision (Supplementary Table S5). The four resequenced clones showed the expected alternative allele for the corresponding SC-SNVs, which is in agreement with the WGR data (Supplementary Table S6).

**Figure 3:**
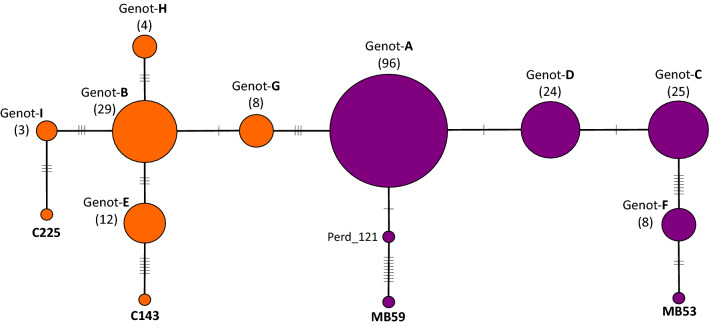
Intra-cultivar clonal genotypic diversity estimated with a custom-designed genotyping chip. A median-joining network was built with the genotypes using 41 SNVs loci obtained for 214 samples. Each circle represents a genotype and its size is proportional to the genotype frequency. We found 14 clonal genotypes, nine were represented by multiple samples (named from A to I) and five were singletons (C143, C225, MB53, MB59 and Perd_121). The hash marks crossing the connecting lines indicate the number of mutational steps differentiating genotypes. The color code represents groups Fr (orange) and Ar (purple).

The genotypes of 214 samples based on 41 SNVs (Supplementary Table S6), constituted the dataset used in the subsequent genetic diversity analyses. We built a median-joining network, and identified 14 different clonal genotypes: five singletons (i.e. genotypes observed only for one sample) and nine genotypes (named A to I) represented by more than one sample (Fig. 3). Most genotypes differentiated from each other by one to three SNVs; except for Genotype-F, C143 and MB59, which accumulated seven, six and nine SNVs, respectively, differentiating these genotypes from the corresponding closest one. Apart from the five singletons, the number of samples represented by each genotype ranged from 96 (Genotype-A) to three (Genotype-I). Genotype-A was the most abundant across our sampling, comprising 45% of the analyzed accessions. After inspecting the origin of the samples, no association was observed between the sampled mass selections and the genotypes assignment, thus indicating that most genotypes had representatives from different mass selections (Supplementary Table S7). The five singleton genotypes corresponded to a sample from Perdriel mass selection (Perd_121) and to the four resequenced accessions. As consequence of the WGR origin of markers included in the genotyping chip, accessions MB53, MB59, C143 and C225 were the most differentiated samples (Fig. 3), due to SC-SNVs that were variable only for them (Supplementary Table S5). After a more stringent analysis, based solely on the 22 SNVs with at least two samples with the alternative allele, the main nine genotypes (A–I) were still retrieved, as well as the singletons C225 and MB53 (Supplementary Fig. S3). In this analysis, C143, MB59 and Perd_121 did not appear as singletons, but were included in genotypes E, F and A, respectively.

**Figure 4:**
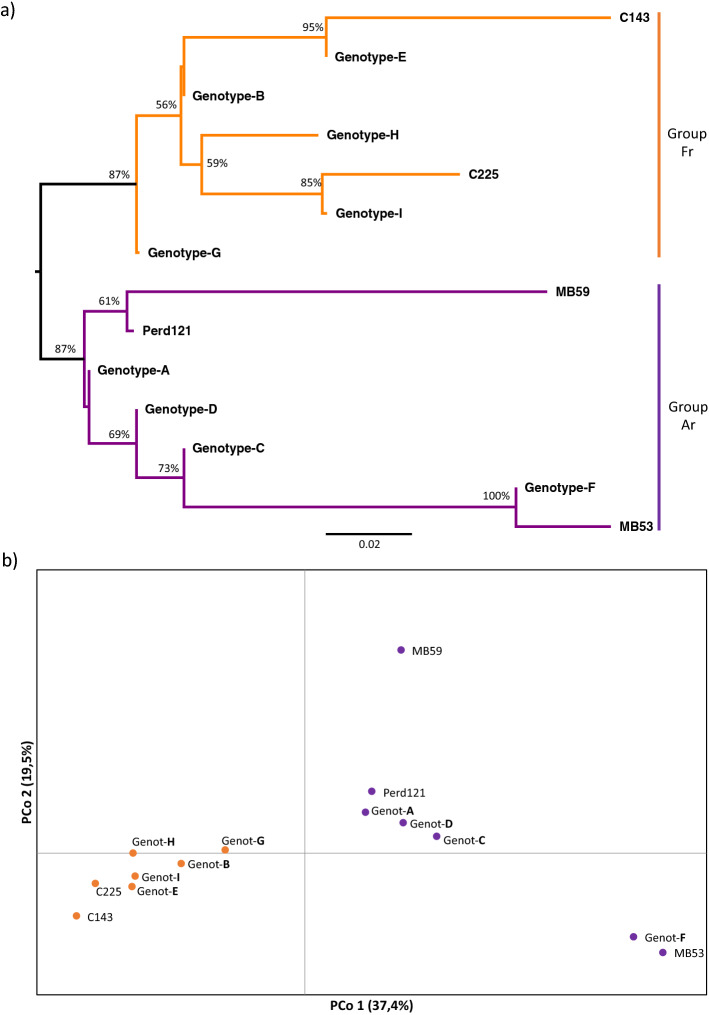
The phylogenetic relationships and genetic distance among the identified genotypes (based on 41 SNVs) suggest the existence of two clonal lineages in Malbec. (**a**) Neighbor-joining tree based on *p*-distances, bootstrap supports values > 50% are shown. The orange clade (Group-Fr) included genotypes closely related to the resequenced accessions that have long remained in Europe (C143 and C225). The purple clade (Group-Ar) included genotypes closely related to the resequenced accessions with more than 70 years of clonal propagation in Argentina (MB53 and MB59). (**b**) Principal coordinates analysis (PCoA) based on genetic distances. PCoA retrieved the same relations as the phylogenetic analyses performed among the genotypes identified, differentiating between Group-Ar (purple) and Group-Fr (orange).

We then studied the phylogenic relations among the 14 clonal genotypes identified on the basis of the 41 SNVs. This analysis included a unique sequence representing each of the nine genotypes (A–I) and the five singletons. The resulting phylogenetic tree displayed the existence of two divergent clonal lineages, named Group-Ar (Argentina) and Group-Fr (France) (Fig. 4a). Group-Ar was driven by the resequenced accessions with > 70 years of clonal propagation in Argentina (MB53 and MB59) and clustered the closely related genotypes A, C, D and F. These genotypes represented the great majority of the samples analyzed (155), including also the singleton genotype Perd_121. On the other hand, Group-Fr was driven by the resequenced accessions that remained for a longer period of time close to the origin of Malbec in France, never grown in Argentina (C143) or with less than 30 years of clonal propagation in Argentina (C225) and clustered the genotypes more closely related to them: E, B, G, H, I. In total, 64 samples clustered in Group-Fr, including all the analyzed samples with less than 30 years of history of clonal propagation in Argentina (Cot42, Cot46, Cot595, Cot596, Cot598, Inta19) or never grown outside Europe (Esp217). Even though Genotype-G was clearly differentiated from other genotypes of Group-Fr (Fig. 4a), all the analyses performed (see below) consistently placed it closer to genotypes from this group. Differences between Group-Ar and Group-Fr were also observed by a principal coordinate analysis (PCoA), in which the PCo1 and PCo2 explained almost 55% of the genotypic variance (Fig. 4b). The separation between the two groups was mainly detected by PCo1 (37.4%). All genotypes with a shorter genetic distance to C143 and C225 clustered together (including Genotype-G); and the same situation was observed for genotypes closely related to MB53 and MB59, although with a greater degree of dispersion (Fig. 4b). The PCoA that was based only on the four Sh-SNVs also allowed distinguishing groups Ar and Fr. Again, Genotype-G was clearly differentiated from the two groups, but still appeared closer to Group-Fr (Supplementary Fig. S4). Finally, the results obtained in the analysis of the molecular variance (AMOVA) indicated that a significant proportion of the total molecular variance was explained after grouping and contrasting genotypes included in Group-Ar and Group-Fr. The highest AMOVA value was obtained when Genotype-G was included in Group-Fr, *Phi*_PT_ = 0,39 (*p* = 0,001).

## Discussion

Grapevines that are currently cultivated (*V. vinifera* ssp. *sativa*) have retained most of the genetic diversity present in their wild counterpart, ssp. *sylvestris*^21,38^. This genetic diversity is evidenced through the great variability observed among cultivars^7,24^. However, as expected, genetic variation is strongly reduced at the intra-cultivar level. In this work we applied a stringent procedure to identify a reliable set of single nucleotide variants, that were employed to build a high throughput genotyping experiment for the assessment of the clonal genetic diversity in Malbec grapevines. We detected several clonal genotypes and found evidence on how human actions might have driven the accumulation of different somatic mutations, ultimately influencing the existence of two clonal lineages.

Somatic mutations mostly accumulate as heterozygous variants, which are more likely to generate false positives in variant calling analyses^39^. Therefore, a major challenge when processing high-throughput genomic data, for clonal genetic diversity studies, is to avoid the overestimation of the number of variants^39,40^. Stringent bioinformatics procedures and experimental corroboration of the called variants might provide more certainty in this regard. Herein, we obtained a set of variants that were consistently called by three different software packages, i.e. GATK^41^, BCFtools^42^ and VarScan2^43^. To further eliminate spurious variants, stringent bioinformatics filters were applied, particularly related to the read edit distance and the variant allele frequency (VAF). Moreover, the experimental corroboration of the identified variants was successfully performed by means of two alternative technologies, i.e. Sanger sequencing (Supplementary Fig. S2) and the Fluidigm genotyping chip (Supplementary Table S6). All tested SNVs showed the expected alternative allele for the expected sample; therefore, the proportion of false positive results among the identified variants could be assumed to be non-significant. This stringent workflow allowed us to obtain a reliable set of SNVs that was employed to custom-design a genotyping experiment, to be applied for the genotyping of Malbec grapevines.

The identified number of SNPs distinguishing Malbec from the PN40024 genotype (Fig. 1), is within the range reported in other works comparing the genetic diversity between a grapevine cultivar and the reference genome^44–46^. On the other hand, we present here the lowest total number of SNVs reported so far for a grapevine cultivar. Previous works studying the intra-cultivar genetic diversity (using WGR data) identified different numbers of SNVs, ranging from the few thousand in Chardonnay^30^ and Nebbiolo^5^, to several thousands in Zinfandel^6^. A reason for the low number of SNVs reported in this work might be that, assuming the presence of putative false negatives in the variant calling procedure, we employed a stringent filtering to yield reliable markers for subsequent identification of genotypes. Nonetheless, comparing the number of variants reported in different works is challenging, because final results might be influenced by technical and biological factors, as well as by the final scope of the analysis^47,48^. We also observed that SNVs affected preferably the intergenic regions (Supplementary Fig. S1), and that, in those regions, transitions are more frequent than transversions (Supplementary Table S2), as reported for Zinfandel^6^. Regardless of the differences in the absolute numbers of SNVs identified for each cultivar, it is evident that clonal genetic diversity in grapevines is scarce and prone to accumulate in intergenic regions. This observation further corroborates the role of clonal propagation and selection in preserving the desired phenotypes of cultivars, while stabilizing the accumulation rate of undesired novel genetic variations^2^.

Despite the low degree of intra-cultivar genetic diversity in grapevines, we were able to design an informative genotyping experiment that allowed distinguishing 14 different clonal genotypes within Malbec (Fig. 3). Nine of the genotypes identified were represented by more than one sample, harboring accessions coming from the different mass selections that were sampled (Supplementary Table S7). Plants from the same mass selection share particular traits of productive interest (data provided by Mercier nursery, Supplementary Table S8). Even though we did not perform a formal phenotypic analysis, we found that the sought phenotypic homogeneity in mass selections contrasts with the observed genetic diversity. On the other hand, regarding the number of samples represented by each of the identified clonal genotypes was highly variable. Genotype-A was the most abundant, including almost half of the accessions studied (Fig. 3). This finding suggests that this has been the most widely propagated genotype in Argentina, either as a consequence of a “bottleneck effect” caused by ancestral introductions of Malbec in South America, and/or subsequent selections potentially favored by its productive performance. However, we cannot rule out the fact that the abundance of Genotype-A could also be due to a methodological artifact. Probably, including more samples with diverse origins, as well as employing additional SNVs markers, will turn into a greater number of genotypes each one represented by fewer samples. Despite these caveats, and even with a reduced set of genetic markers reported here, it was still possible to retrieve the main nine genotypes (Supplementary Fig. S3).

The genotypes identified clustered in two genetically divergent clonal lineages, named groups Ar and Fr (Fig. 4a,b). Moreover, by employing solely the four Sh-SNVs included in the chip was enough to detect these groups (Supplementary Fig. S4). The two clonal lineages identified in cultivar Malbec could be reflecting the effect of anthropically driven actions, operating in combination with natural biological processes. The only natural source of genetic variation in grapevine cultivars are the somatic mutations and epimutations, which arise during vine growth and might be passed to daughter vines through vegetative propagation^49,50^. Therefore, shared mutated positions constitute fingerprints that provide information on the past history of a given clonal genotype^51^. At the same time, as a crop species, human actions such as plants transportation from one country to another, clonal propagation and clonal selection, play a major role on the observed patterns of genetic diversity^2,22,49^.

Historical records indicate that the first Malbec plants were introduced from France to Argentina (Mendoza province) in the 1850s^32,34^. After that first event, wine producers introduced plants to Argentina at a continuous rate, that was slightly increased during the 1990s^52^. The two clonal lineages identified could be reflecting this particular history of plants movements from France to Argentina, as well as the subsequent clonal propagation under different environmental conditions and alternative selection criteria on both sides of the Atlantic Ocean. Groups Ar and Fr might be allowing us to distinguish between genotypes that have gone through alternative pathways and accumulated different somatic mutations, while bringing together those genotypes with a recent shared history. More precisely, genotypes included in Group-Fr are closely related to the resequenced accessions (C143 and C225) that have longer remained in Europe and close to the origin of this cultivar in France. Group-Fr also included all the other genotyped samples that have never been grown in Argentina, or were introduced in this country over the past 30 years. On the other hand, genotypes from Group-Ar are closely related to the resequenced accessions with a longer time span of clonal propagation in Argentina (MB53 and MB59), suggesting a closer link to those first plants introduced from France during the 1850s. Among the analyzed samples, we can easily identify those that have never been grown or which have recently been introduced in Argentina (< 30 years). However, we cannot accurately determine the exact time span of clonal propagation for those accessions that have remained for more than 70 years in Argentina. In particular, some of the latter accessions were included in Group-Fr (Supplementary Table S7), indicating possible traceability inconsistencies. For accessions included in Genotype-G, intermediate times of introduction in Argentina could be suggested, since this genotype clustered in Group-Fr but appeared clearly differentiated within it (Fig. 4a). Moreover, it was not possible to trace back the precise propagation records of individual plants from the sampled mass selections; the only available information is related to the vineyard of origin and production criteria of selection. In this context of uncertainty, it is important to highlight that, by means of the designed genotyping experiment, we were able to retrieve the same phylogenetic relations observed among the four resequenced accessions with a known history.

The set of markers included in the chip proved useful for the identification of clonal genotypes and unveiled the existence of two clonal lineages. In fact, genotyping as few as four Sh-SNVs would be enough to determine if a Malbec plant is closely related, either to ancestors that were early introduced in Argentina or to those that remained longer in Europe. SC-SNVs were also essential for the detection of all the reported clonal genotypes. This observation supports the importance of combining single clone and shared variants to enhance the sensitivity of genotyping methods in clonal genetic diversity studies^30,51^. In this sense, custom genotyping for grapevine cultivars has proved to be a valuable tool with applications that are also relevant for the wine industry; including nurseries, wineries and producers. For instance, a WGR genotyping approach was employed in Chardonnay cultivar to fill in the historical gaps of commercially relevant clonal accessions^51^. On the other hand, in Nebbiolo cultivar TaqMan genotyping has proved useful for clonal geographic traceability^5^ and for wine authentication purposes^53^.

In this work we set-up a stringent workflow to identify a reliable set of SNVs that were employed to design an informative genotyping chip. We were able to detect several clonal genotypes within Malbec, and provided evidence on the processes that might have driven the accumulation of different somatic mutations, influencing the existence of two clonal lineages. Our findings further support the usefulness of high-throughput genotyping methods in grapevines to better understand cultivars’ history, and as a valuable tool for the wine industry. Future studies should determine if the identified clonal lineages in Malbec could be associated to phenotypic differences affecting traits of productive interest.

## Materials and methods

### Biological material

To perform whole genome resequencing, we obtained young leaves and shoot tips from four Malbec clonal accessions. Two accessions, Malbec-501 (MB53) and Cot-ENTAV-598 (C225), were sampled at Mercier Argentina nursery (hereafter: Mercier) collection (Perdriel, Lujan de Cuyo, Mendoza). The other two accessions, Malbec-059 (MB59) and Cot-143 (C143), were sampled at Mercier Granata vineyards and “Finca El Encín” ampelographic collection (Alcala de Henares, Spain), respectively. The two accessions labeled as Malbec (MB53 and MB59) represent plants with long history of clonal propagation in Argentina, meaning that they have been propagated in this country for more than 70 years (Mercier records). We also included two accessions labeled as Cot (C225 and C143), having short and null histories of clonal propagation in Argentina. More precisely, C225 was introduced into Argentina from France (ENTAV-INRA) during the 1990s (Mercier records). C143 arrived to “Finca El Encin” in 1951 from INRA Vassal-Montpellier (France), and has never been grown in Argentina.

For the genotyping analysis, shoot tips and young leaves were obtained from 219 plants*.* We sampled 70 Malbec clonal accessions (including the four resequenced ones) belonging to: (a) the National Institute of Agricultural Technology (INTA Mendoza) collection (28 clones), (b) Mercier collection (37 clones), (c) Mercier Granata vineyards (three clones) and (d) “Finca El Encin” (two clones). The time spans of clonal propagation in Argentina were obtained from INTA Mendoza and Mercier records. We also obtained 30 samples from five different Mercier mass selections (150 samples in all), located at Granata vineyards. Further details about mass selections and sample origin is available in Supplementary Tables S8 and S9, respectively. All samples analyzed in this work were used with the explicit consent of the respective owner company (Vivero Mercier Argentina) and public institutions (INTA Mendoza and IMIDRA “Finca El Encin”).

### Whole genome resequencing, variant calling and validation

#### DNA extractions and resequencing

Whole genomic DNA from the four Malbec accessions (MB53, MB59, C225 and C143) was isolated using the DNeasy Plant Mini Kit (Qiagen), including an RNase treatment according to manufacturer’s recommendations. DNA quantification and quality checks were performed with a NanoDrop 2000 spectrophotometer and 5% agarose gel electrophoresis. The preparation of the library and sequencing were performed at the Center for Genomic Regulation (Barcelona, Spain). Paired-end reads 125 bp long were generated using the HiSeq 2000 Illumina technology with the Sequencing v4 chemistry.

#### Reads alignment, variant calling and filtering

Standard quality checks of *fastq* files were performed with FastQC^54^. Raw reads were pre-processed following the GATK Best Practices workflow with the GenomeAnalysisTK-3.3-0 toolkit^41^. After marking Illumina adapters with Picard toolkit v2.9.4^55^, sequences were aligned to the *Vitis vinifera* L. reference genome PN40024^4^. We employed the Burrows–Wheeler algorithm as implemented in BWA-MEM v0.7.12-r1039^56^ to align our reads to the reference genome. Mapped reads were thoroughly filtered also with the Picard toolkit^55^ allowing only non-duplicates, unique and concordant alignments with a maximum read edit distance of 1 per 25 nucleotides of query sequence^57^. Filtered alignments were used as input for variant calling, using three different tools operating in the multi-allelic mode and with default parameters: GATK UnifiedGenotyper^41^, BCFtools call v1.9^42^ and and VarScan2 mpileup2cns v2.3.9^43^. gVCF files generated for each accession were intersected and only those SNVs identified by all three callers were retained, while INDELs and structural variations were not considered in this study**.** Bioinformatics procedures were adjusted using a set of SNPs between Malbec and PN40024, retrieved from Vitis18kSNP array results^58^. Only confident identified raw variants were retained on the basis of WGR recommendations of total depth (DP), variant allele frequency (VAF), strand bias and distance bias (Bentley et al. 2008). Cut-off values for these parameters were: DP = [15–150], VAF(Ref) ≤ 0.025, VAF(Het) = [0.25–0.75], VAF(Hom) ≥ [0.95]. Significance levels were: P-value (strand bias) ≤ 0.0001 and P-value (distance bias) ≤ 0.0001. Variant allele frequency ranges were particularly adjusted to reduce—at the minimum possible—the presence of spurious variants. Chimeric mutations are frequent in grapevines, occurring differentially between the L1 and L2 cell layers of the developmental tissue from the apical meristem^49^. The L1 layer gives rise to the epidermis and represents a smaller proportion of the total tissues conforming a plant (nearly 30%)^50^. With the VAF filters employed we expected to detect most of the chimeric mutations occurring in the L2 cell layer (half of the total frequency in 60% of somatic tissues). Whereas chimeric heterozygous mutations occurring only in the L1 would be mostly excluded. With the aim of reducing the number of false positive results, we assumed these variants loss as a trade-off. The distribution of variants across the genome, the genomic regions affected by these variants and the transitions to transversions ratio were estimated with SnpSift^59^.

#### Corroboration of the bioinformatics pipeline

We employed IGV v2.3.97^60^ to manually corroborate a subset of the SNVs identified and to isolate a ~ 600 bp sequence containing the target SNVs in the mid-region. These sequences were used as templates for primer design to perform PCRs and Sanger sequencing of amplicons. In order to avoid both, primer annealing and later genotyping problems, we checked for the absence of variable sites on the 5′- and 3′-regions of the sequence and in the proximities of the SNV target position. Primers were designed using the Primer BLAST tool^61^ with an average annealing temperature of 60.3 °C (range 58.8–62.5 °C) and an average amplicon length of 447 bp (range 300–582 bp). More details are available in Supplementary Table S3. PCRs were carried out in a 25 μl final reaction volume containing 0.3 µl (5 U/μl) High Fidelity Taq Polymerase (TransTaq), 1.25 µl (10x) buffer GC-enhancer (TransTaq), (2.5 µl) 10 × PCR buffer I (TransTaq), 1 µl (2.5 mM) dNTPs, 1 µl of each (10 μM) reverse and forward primer and 3 µl (40 ng/μl) DNA template. PCR cycles consisted of a denaturation step of 5 min at 98 °C, 35 cycles of 3 s at 94 °C, 30 s at 60 °C and 30 s at 72 °C, and final extension period of 7 min at 72 °C. PCR products were purified using the ExoSAP-IT PCR Product Cleanup kit (Thermo Fisher Scientific) following the manufacturer’s recommendations. To validate the target SNVs, electropherograms of the four resequenced clones were aligned and inspected with Codon Code Aligner v4.0.4 (CodonCode Corp. USA). SNVs were considered validated if the allelic state at the position of interest in the sequence coincided with that observed in the *vcf* file and in the IGV genome browser. For example, for a particular heterozygous SC-SNV, the clone for which the variant was identified must be heterozygous and for the other three clones must be homozygous as the reference genotype for the target position.

### Genotyping

DNA extractions were performed employing the NucleoSpin Plant II Mini kit (Macherey–Nagel). The quantification of the isolated DNA was performed using NanoDrop 8000 Spectrophotometer (Thermo Fisher Scientific) and the Qubit 2.0 Fluorometer (Invitrogen, Life Technologies).

To build the genotyping chip, only heterozygous alternative variants were selected from the deep-filtered list. We included 42 SC-SNVs and 6 Sh-SNVs, based on their capacity to discriminate among the four resequenced clones. For the SC-SNVs, equivalent number of variants for each clone were chosen. Sh-SNVs were picked for their capacity to differentiate between the resequenced accessions with a long history of clonal propagation in Argentina and those with a short or null history of propagation in this country. The latter was because we were particularly interested in identifying genetic markers that could consistently resemble that historical aspect across our samples. The SNVs chosen were distributed across different chromosomes to better represent the genomic diversity. In total, 48 sequences containing each SNV of interest (Supplementary Table S4) were provided to the Genomics Service Sequencing and Genotyping Unit (UPV/EHU) (Bizkaia, Spain), to design probes to build a Fluidigm chip (https://www.fluidigm.com/) and perform the genotyping analyses. Each experiment allowed the simultaneous genotyping of 48 samples using 48 SNVs in a two-step reaction. In the first step, the target region containing the position to be genotyped is amplified using two pre-amplification primers (locus-specific primer and target-specific amplification). In the second step, an additional PCR amplifies a portion of that SNVs target region using the locus-specific primer and two fluorescent allele-specific primers, which are internal primers containing either the first or the second allele, respectively. Finally, the genotype is determined by measuring the fluorescence intensity of both alleles using the Fluidigm genotyping analysis software.

### Clonal genetic diversity analyses

To assess the degree of genetic variation among Malbec clones, biallelic genotypes were coded to sequences in the *fasta* format. Firstly, we performed a phylogenetic analysis with the MEGA v7.0.26 software^62^ including only the four resequenced clones and using the PN40024 genotype as outgroup. A neighbor-joining tree using uncorrected *p*-distances was estimated using the deep-filtered list of SNVs, support values for the nodes were obtained after 200 bootstrap iterations.

To assess the degree of diversity across all the genotyped samples a median-joining network^63^ analysis was performed with PopART^64^ to identify the number of different genotypes, their frequencies and their phylogenetic relations. We then obtained a single representative sequence for each of the genotypes identified to reconstruct a neighbor-joining tree employing MEGA v7.0.26^62^, using the same parameters described above. The genetic diversity was also analyzed considering each SNVs position as an independent marker, and by estimating codominant genotypic distances among the identified genotypes with GenAlEx v6.5^65^. Genetic distances among genotypes were analyzed with a model-free approach of principal coordinates analysis (PCoA) to detect potential groups of genotypes closely related to each other. Finally, an AMOVA was performed to estimate the proportion of the total molecular variance that could be explained by the variance observed between groups. We obtained the *Phi*_PT_ parameter recommended for distances obtained from co-dominant genotypic data. The *p-*value was obtained after 900 bootstrap iterations. Both PCoA and AMOVA were also performed with the GenAlEx v6.5 software.

## Supplementary Information

Supplementary Information 1.

Supplementary Information 2.

Supplementary Information 3.

Supplementary Information 4.

Supplementary Information 5.

Supplementary Information 6.

## Data Availability

SRA files containing raw genomic data for the four resequenced Malbec clones is available at NCBI, BioProject: PRJNA663442.
