# Supplementary material for: Whole genome resequencing and custom genotyping unveil clonal lineages in ‘Malbec’ grapevines (Vitis vinifera L.)
Source: Sci Rep. 2021 Apr 8;11:7775. doi: 10.1038/s41598-021-87445-y (PMC8032709; doi:10.1038/s41598-021-87445-y)
Supplement: Supplementary file 1 — Supplementary Information 1. [file 41598_2021_87445_MOESM1_ESM.pdf]

Whole genome resequencing and custom genotyping unveil clonal lineages in 'Malbec' grapevines (*Vitis vinifera* L.)

Luciano Calderón, Nuria Mauri, Claudio Muñoz, Pablo Carbonell-Bejerano, Laura Bree, Daniel Bergamin, Cristobal Sola, Sebastian Gomez-Talquenca, Carolina Royo, Javier Ibáñez, José Miguel Martínez-Zapater & Diego Lijavetzky

Supplementary figures

Figure S1. Variants and effect type distribution across the genome for clonal genetic variations (941 SNVs) and for variants distinguishing Malbec from PN40024 (2.1 million SNPs), as provided by SNPeff.

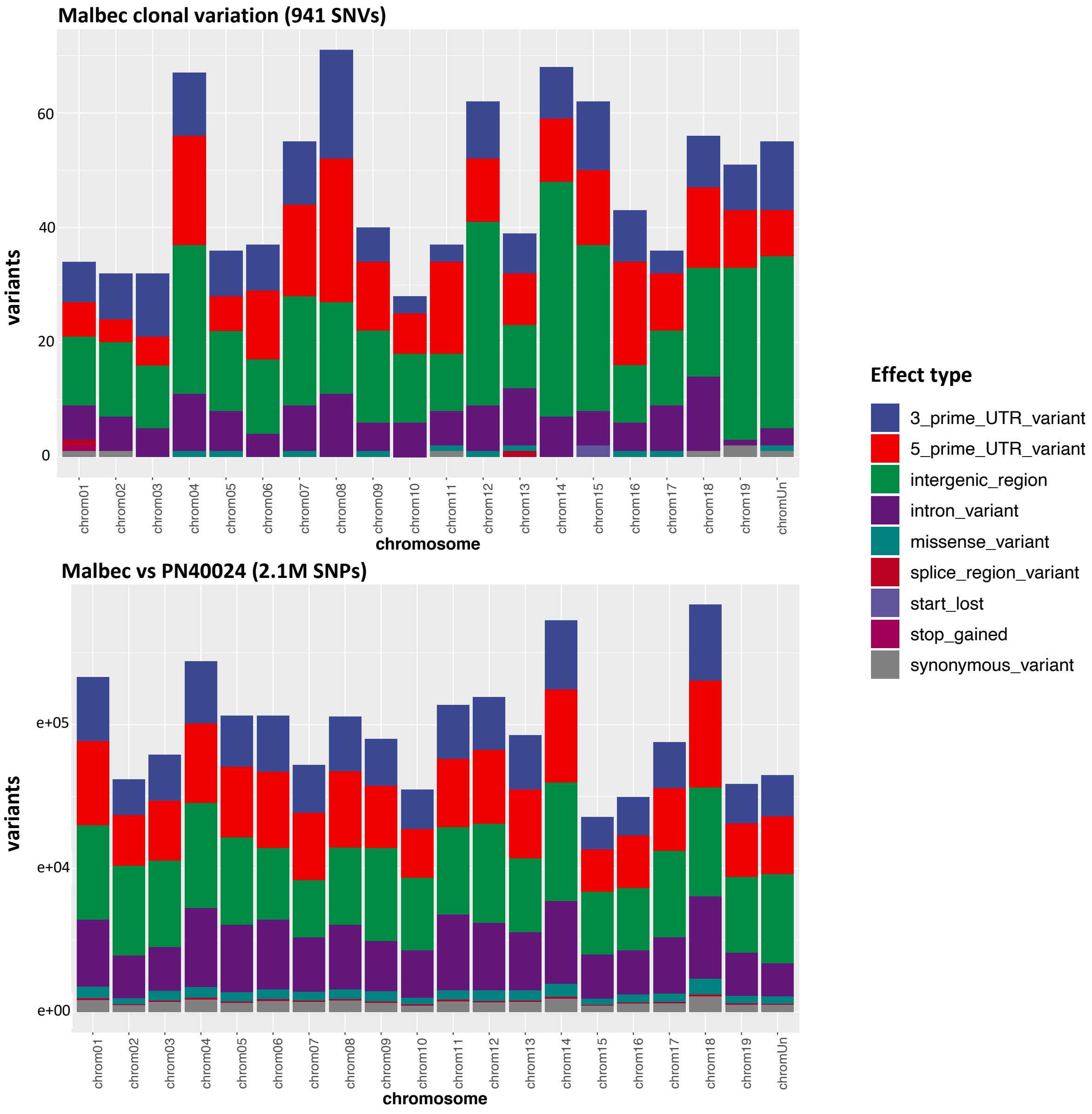

Figure S2. Example of four SC-SNVs validated through Sanger sequencing, one for each of the four re-sequenced clones. Electropherograms show that the position of interest (indicated with the blue line) is heterozygous alternative for the expected clone (on top) and homozygous as the reference in the other three.

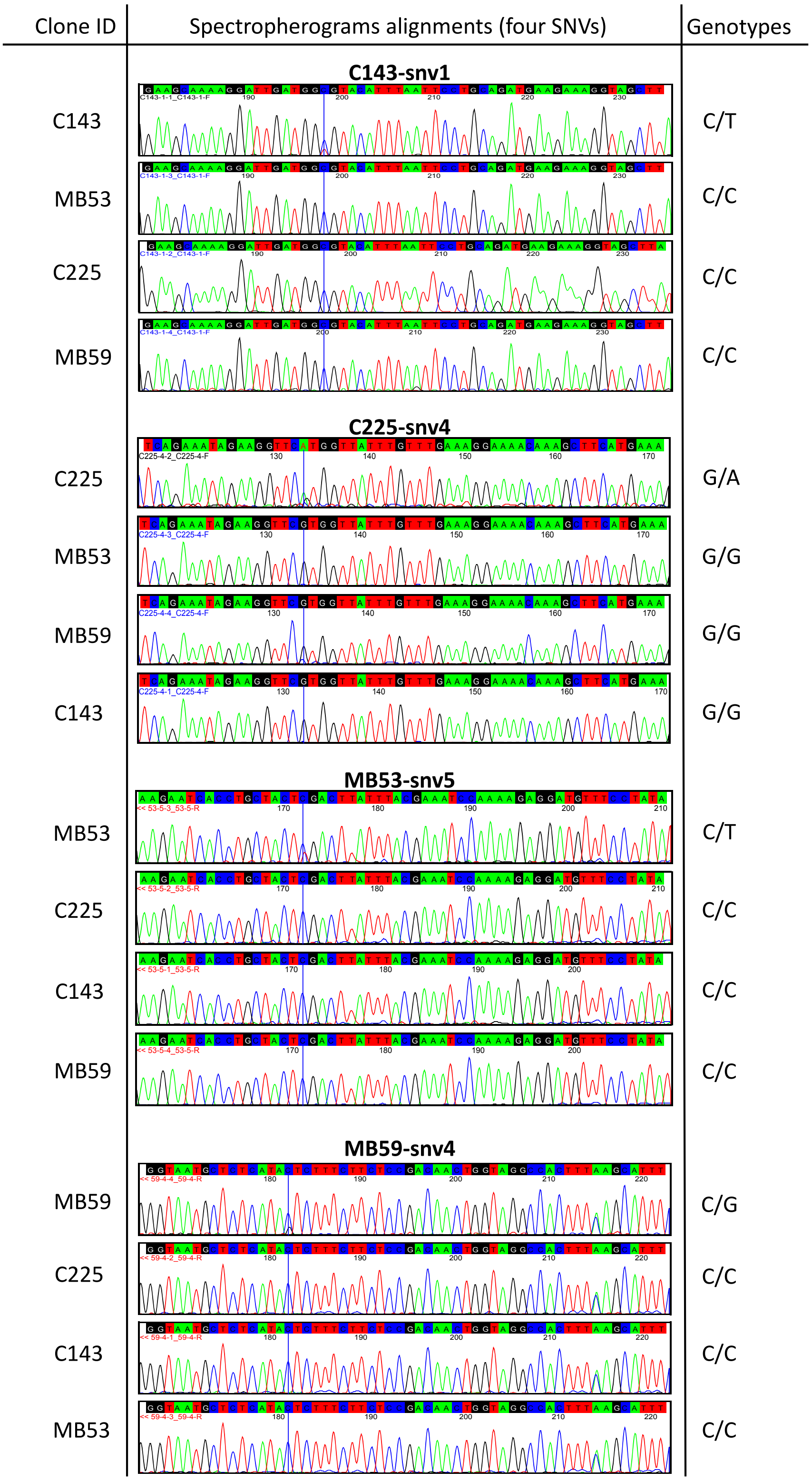

Figure S3. Median joining network based on the 22 SNVs that showed more than two samples with the alternative allele. We recovered the main nine groups of genotypes than the analysis using the 41 SNVs, but only two singleton genotypes: C225 and MB59.

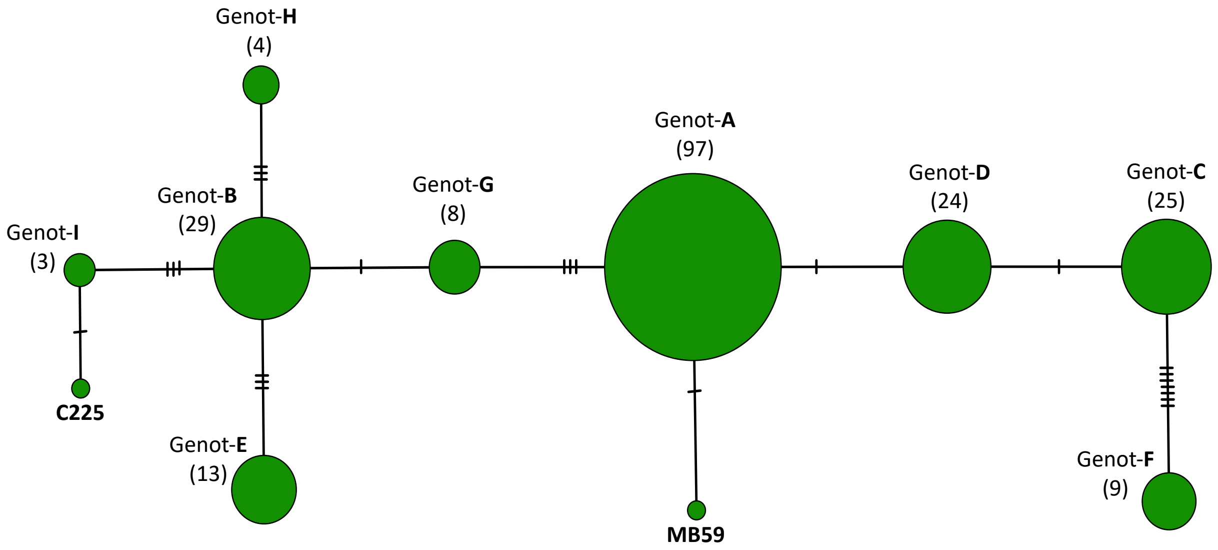

Figure S4. Principal coordinates analysis based only on the four shared SNV (Sh-SNV). PCoA recovers the distinction between the two clonal lineages (Groups Ar and Fr).

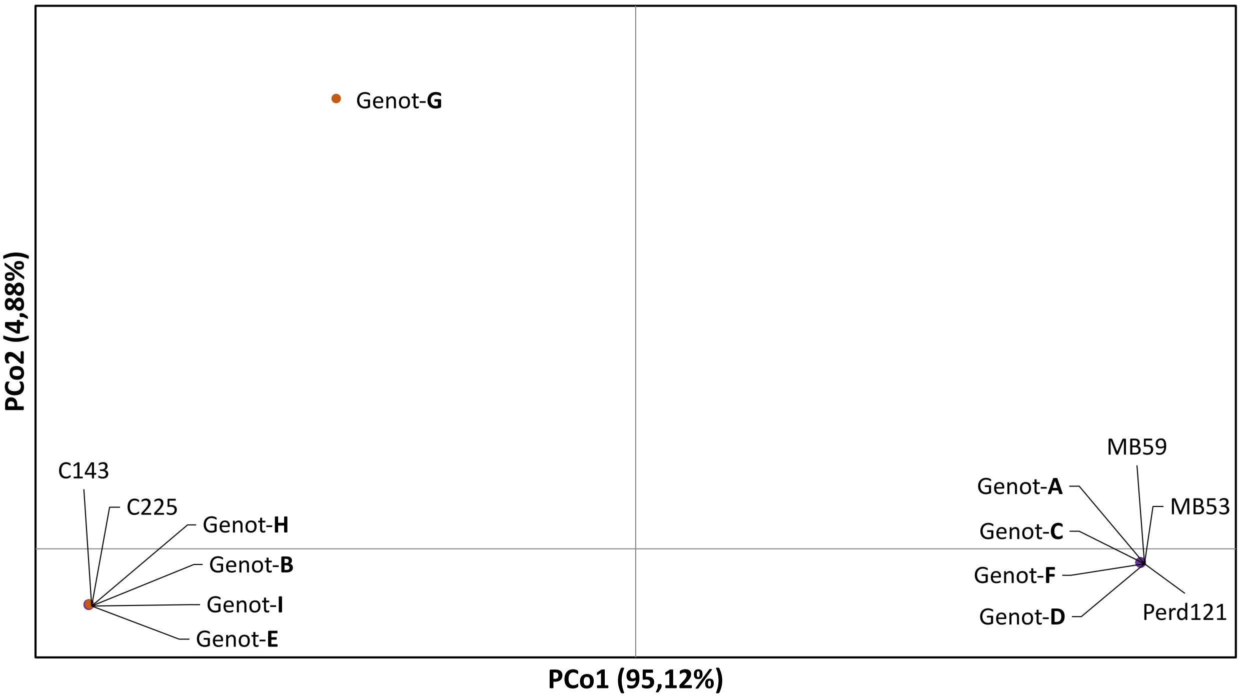

Supplementary Tables

Table S1. Raw data of paired-end (PE) reads obtained for each resequenced clone. Alignment stats values reported after filtering, percentage and coverage of reads alignment to *Vitis vinifera* L. reference genome (PN40024).

	Raw data			Alignment stats	
Clone ID	PE reads obtained	Reads size	Total sequence (Gb)	Covered %	Depth coverage (+SD)
C143	86,859,172	125	10.85	77.55	28.47 (70.66)
C225	94,388,045	125	11.79	77.9	30.26 (59.52)
MB53	90,054,921	125	11.25	78.01	31.29 (48.7)
MB59	90,125,307	125	11.26	77.94	29.96 (40.61)

Table S2. Genomic context of variants occurrence for each of the resequenced clones and for SNPs shared by the four clones. Variants occurring in intergenic, intronic and exonic regions, and the transitions (Tr) to transversions (Tv) ratio for those regions are shown.

	Counts			Tr/Tv ratio		
IDs	Intergenic	Intron	Exon	Intergenic	Intron	Exon
C143	451	133	11	2.55	2.26	10
C225	439	116	8	2.54	2.25	7
MB53	440	117	8	2.64	2.07	7
MB59	447	140	10	2.73	2.06	4
Malbec vs PN40024	1,179,296	607,119	134,727	2.39	1.86	2

Table S5. Details of the 41 SNVs employed in the final analyses. The name of the SNV refers to the re-sequenced clone for which it was identified. Genomic position is relative to PN40024, as well as the reference allele (Ref) state. The alternative allele (Alt) was observed in that position only for one of the four clones. Finally, the genotypic frequencies for each SNV based on the 214 successfully genotyped samples.

		Allelic state		Genotypic frequencies		
SNVs-ID	Genomic position	Ref	Alt	XX	XY	YY
C143-snv1	chr10:2146290	C	T	213	1	0
C143-snv2	chr10:2250888	C	T	201	13	0
C143-snv3	chr11:2533840	T	C	213	1	0
C143-snv4	chr12:5997741	T	C	213	1	0
C143-snv6	chr14:12612349	C	G	201	13	0
C143-snv7	chr16:18527058	T	C	213	1	0
C143-snv11	chr7:5723085	G	A	201	13	0
C143-snv13	chrUn:8135037	C	T	213	1	0
C143-snv14	chrUn:30681019	G	A	213	1	0
C225-snv1	chr11:13104147	G	A	213	1	0
C225-snv4	chr15:1260006	G	A	209	1	4
C225-snv6	chr18:18692633	T	C	213	1	0
C225-snv8	chr19:15624611	G	T	213	1	0
C225-snv9	chr4:17654346	G	A	210	4	0
C225-snv11	chr6:10576840	G	A	210	4	0

C225-snv15	chrUn:1879577	C	T	210	4	0
MB53-snv1	chr1:8947277	G	A	205	9	0
MB53-snv2	chr12:11248831	C	T	213	1	0
MB53-snv3	chr17:8192917	G	A	213	1	0
MB53-snv4	chr17:14078937	G	A	180	34	0
MB53-snv5	chr18:2701946	C	T	205	9	0
MB53-snv6	chr19:9264852	C	T	205	9	0
MB53-snv7	chr4:18276730	A	C	180	34	0
MB53-snv8	chr6:16432984	A	G	205	9	0
MB53-snv9	chr7:10631864	C	T	205	9	0
MB53-snv10	chr7:16870565	C	G	205	9	0
MB53-snv11	chrUn:1602277	G	A	205	9	0
MB59-snv1	chr1:4231365	G	A	213	1	0
MB59-snv2	chr10:9009503	C	A	209	5	0
MB59-snv4	chr14:612099	C	G	213	1	0
MB59-snv5	chr14:25250166	C	T	213	1	0
MB59-snv9	chr17:8058225	C	G	212	2	0
MB59-snv10	chr18:9067411	T	C	213	1	0
MB59-snv11	chr19:15786950	C	T	213	1	0
MB59-snv12	chr4:8993951	A	G	213	1	0
MB59-snv14	chr9:5534019	T	C	213	1	0
MB59-snv15	chr9:18487093	T	C	213	1	0
MB53-MB59-snv2	chr17:161911	C	T	50	164	0
MB53-MB59-snv3	chr19:22916577	C	T	58	156	0
MB53-MB59-snv5	chr5:22788880	C	G	58	156	0
MB53-MB59-snv6	chrUn:14723119	C	T	58	156	0

Table S8. Brief description of the five sampled 'Malbec' mass selections. Information provided by Mercier Argentina nursery.

Mass selection ID	Vineyard origin	Plants selection criteria	Selection outcome
Perdriel	District: Perdriel<br>Department: Luján de Cuyo<br>Province: Mendoza	Absence of virus and fungus.<br>Absence of *millerandage.*<br>Loose cluster, medium size grape, red rachis at maturity.	Average Productivity: 155 qq/Ha<br>Average cluster weight: 120 gr<br>Average total polyphenols index (IPT) = 60.
Vista Flores	District: Vista Flores<br>Department: Tunuyán<br>Province: Mendoza	Absence of virus and fungus.<br>The complete vineyard was preserved because of high. quality wines production.	Average productivity: 170 qq/Ha<br>Average cluster weight: 190 gr<br>Average total polyphenols index (IPT) = 87.
Las Compuertas	District: Las Compuertas<br>Department: Luján de Cuyo<br>Province: Mendoza	Absence of virus and fungus.<br>Absence of *millerandage.*<br>Low productivity and high-quality wine production.	Average productivity: 110 qq/Ha<br>Average cluster weight: 90 gr<br>Average total polyphenols index (IPT) = 50.
Las Paredes	District: Las Paredes<br>Department: San Rafael<br>Province: Mendoza	Absence of virus and fungus.<br>Medium size grape and cluster, red rachis at maturity.	Average productivity: 193 qq/Ha<br>Average cluster weight: 110 gr<br>Average total polyphenols index (IPT) = 45.
Cobos	District: Agrelo<br>Department: Lujan de Cuyo<br>Province: Mendoza	Absence of virus and fungus.<br>Grapes skin showing intense purple coloration.	Highly variable productivity: ranging between 160 and 317 qq/Ha.<br>Average cluster weight: 150 gr<br>Average total polyphenols index (IPT) = Not Available
